# First presentation of guttate psoriasis triggered by acute tonsillitis

**DOI:** 10.11604/pamj.2014.17.273.421

**Published:** 2014-04-14

**Authors:** Theocharis Koufakis, Ioannis Gabranis

**Affiliations:** 1 Department of Internal Medicine, General Hospital of Larissa, Larissa, Greece

**Keywords:** Guttate psoriasis, tonsillitis

## Image in medicine

We present a case of a 33 years old man who presented to the Emergency Department of our Hospital with small, red, drop-like lesions located at his arms, legs, torso and back. He had no history of any dermatological disease, but he had a positive maternal family history for plaque psoriasis. The patient had recently recovered from acute tonsillitis after receiving amoxicillin - clavulanic acid therapy. The diagnosis of guttate psoriasis was clinically established, based on the typical presentation and the history of recent upper respiratory infection. He was treated with a combination of oral and topical corticosteroids and he was advised to have frequent follow-up visits to our Dermatology Department. Guttate psoriasis (also known as “Eruptive psoriasis”) is more common in young adults and often follows a bacterial or viral infection, but it has also been associated with drugs, stress, skin injury and other trigger factors.

**Figure 1:**
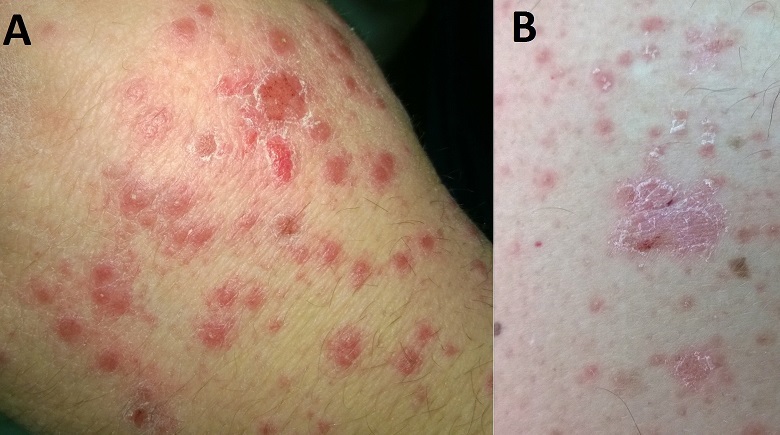
Small, red, drop-like lesions of Guttate psoriasis located at the hand (A) and the arm (B) of the patient

